# Comparative study of the extrinsic properties of poly(lactic acid)-based biocomposites filled with talc *versus* sustainable biocarbon†

**DOI:** 10.1039/c9ra00034h

**Published:** 2019-02-26

**Authors:** Michael R. Snowdon, Feng Wu, Amar K. Mohanty, Manjusri Misra

**Affiliations:** School of Engineering, Thornbrough Building, University of Guelph 80 South Ring Rd E Guelph Ontario Canada N1G 1Y4 mmisra@uoguelph.ca mohanty@uoguelph.ca snowdonm@uoguelph.ca; Bioproducts Discovery & Development Centre (BDDC), Department of Plant Agriculture, Crop Science Building, University of Guelph 117 Reynolds Walk Guelph Ontario Canada N1G 1Y4 fengwu@uoguelph.ca

## Abstract

This study investigates the effects talc and two sizes of biocarbon have as fillers in a PLA bioplastic, when considering them for durable composite applications. Analysis of the PLA-based biocomposites' resistance to wear and flammability accompanied by the vapor barrier characteristics were conducted, with subsequent rheological and thermal properties to further explain the observed results. The compression molded sheets showed a reduction in abrasion by greater than 69% for either filler type compared to the neat PLA due to their high stiffness. In contrast, only the talc provided barrier properties that hindered both water and oxygen permeability, while biocarbon did not possess a high aspect ratio to form a tortuous path necessary for barrier improvement. Yet, the biocarbon-filled PLA biocomposites provided superior flammability resistance due to its char-like caricature that superseded the neat PLA and talc variant which both failed the horizontal burning test. The rheology of the composites provided evidence in the degradation of the PLA chains from the presence of the biocarbon that did not occur with the talc, which may have also contributed to the lower barrier and higher burn resistance from increased dripping. Thus, both talc and biocarbon have their own potential applicability when it comes to acting as a barrier enhancer or flammability retardant due to their intrinsic nature, but both possess wear reinforcement for focus in the tribological area.

## Abstract 2

PLA composites containing sustainable biocarbon providing wear resistance and flame-retardant capabilities on par or superior to conventional talc filler.

## Graphical abstract

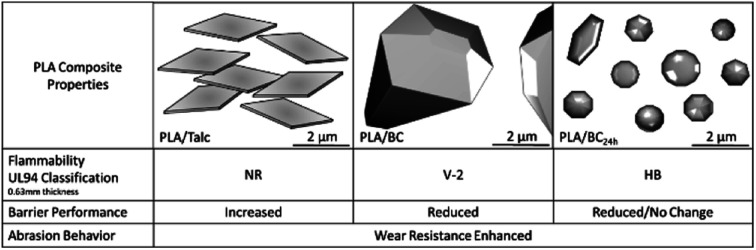

## Introduction

As poly(lactic acid) (PLA) bioplastic remains a suitable option as a biobased and compostable polymer for the thermoplastic industry, there are still avenues in which the material is being explored for more expansive applications. Unlike the common areas of focus including single-use items and short-term packaging, there is potential for its adoption in composite formulations for more specialized applications from automotive parts, electronics, durable goods and long-term packaging.^1^ The shortcomings of PLA lie with the reduced thermo-mechanical stability, poor toughness and limited gas barrier properties,^2^ that ultimately narrows the end-use where biodegradability or bio-content are favored. The use of blending techniques or copolymerization is one method that is used to alleviate these concerns.^3^ However, there has been adoption of including filler variants into PLA to act as an economic and industrial viable solution by lowering the cost of the composite while strengthening targeted properties from dimensional stability, strength or stiffness, permeability and flame retardance features.^4^

Over the past decade, there has been an array of micron to nanosized fillers with or without functionality that have been blended into PLA to form various composites.^5^ It has been found that the utilization of mineral fillers provide beneficial options when well dispersed in the PLA matrix.^6^ These types of materials are appealing to manufacturers of highly demanding composites that require thermal and fire-resistant characteristics that are found in electronic and automotive components.

Still, PLA is not well suited on its own as a flame-resistant polymer as it drips under combustion.^7^ Therefore, similar to the pathways adopted for engineering plastics, PLA can be customized for flame retardancy among other important aspects such as wear resistance and vapor barrier. For example, the adhesion of the matrix and filler play a critical role when improving abrasive wear performance through compatibilization or surface modifications.^8^ These interfacial interactions between polymer and filler are also relevant for enhancing the particulate dispersion throughout the matrix that can provide good barrier properties.^9^ Nevertheless, the general additives or compatibilizers that enable the enhancement of these properties can provoke the degradation of the polyester matrix and weaken the mechanical and thermal stability, causing consideration when having a final application in mind.

One of the frequently investigated reinforcing fillers in PLA is talc, with the ability to impart good dispersion throughout the matrix for barrier, mechanical and thermal optimization.^10^ Likewise, talc can act as an inert filler to provide fire retardant capabilities.^11^ This mineral filler has also been explored for its abilities to prevent abrasive wear in PLA.^8^ Therefore, looking at alternatives to talc to reduce density concerns and provide a more sustainable filler variant, biocarbon has begun to show promise as an inexpensive bioderived additive. In previous work, biocarbon has been incorporated into PLA with an enhancement in the rigidity of injection molded samples without compromising the thermal–mechanical properties.^12^ Other researchers have reported greater wear resistance for PLA 3D-printed biocomposites containing biocarbon from the stiffness it provides.^13^ From these findings, biocarbon demonstrates traits that can enable its use as a multi-purpose filler. Though considerations on the type of biomass-derived carbon used for a specific application need to be chosen based on most biocarbons having a lower carbon content, smaller surface area, larger primary unit particle size and reduced electrical conductivity at low loadings from being a combination of amorphous and turbostratic crystallites over other carbonaceous fillers such as graphene and carbon black.^14,15^

To date, a large degree of the literature has focused on the effects of talc, clays, and micro or nanofillers, when examining the composites for their potential in the areas of hard packaging, electronic casings and interior car applications. In this work, the aim was to evaluate the utilization of PLA composites through the comparison of a conventional filler, talc, *versus* biocarbon variations without any additional compatibilization. Formulations were prepared *via* melt-compounding and compression molding technologies for analysis of the performance characteristics of this biobased filler and its effect on the abrasive, flammability and barrier performance, in formulating a baseline of the benefits and drawbacks biocarbon has on its inclusion in a PLA matrix relative to the commonly utilized talc filler.

## Experimental

### Materials

The general purpose grade Ingeo biopolymer 4043D poly(lactic acid) (PLA), was supplied by NatureWorks, and utilized as the matrix for all composites. While the particulate fillers included, Mistron Vapor R talc from Imerys Talc, with an average particle size of 2 μm, and biocarbon produced from pyrolyzed *Miscanthus* at 650 °C provided by Competitive Green Technologies (Leamington, ON), having two size variations. One obtained *via* an industrial hammer milled process and ground to a size of ≤400 μm with a mean particle range of 20–75 μm (ref. 16) having irregular particle shapes with sharp edges that retain the porous cellular structure of the original grass, and a finer form prepared from a secondary step using the pre-ground biocarbon followed by industrial ball milling for 24 hours, reaching particles <5 μm and a mean particle size of 0.9 μm that is more spherical in form.^17^

### Specimen preparation

The PLA composites were prepared by means of melt-blending in a co-rotating twin screw Xplore MC 15 micro compounder (Netherlands). The PLA was pre-dried in an oven at 80 °C for 4 hours until a moisture of <0.2%, while the biocarbon was pre-dried in an oven at 105 °C for 48 hours to reach a moisture content ∼1.5% and the talc was used as received (0.5% moisture). The melt temperature during extrusion was set at 180 °C with a 100 rpm screw speed and a 2 minute mixing time, before strands were collected. The blends included neat PLA, along with the composite samples with 10 wt% loading of either talc, hammer milled biocarbon (BC), or ball milled biocarbon (BC_24 h_), further denoted PLA, PLA/talc, PLA/BC, and PLA/BC_24 h_, respectively. For the sheet formation, the strands of the resultant material were then pelletized using a strand pelletizer Scheer SGS25. The pellets were prepared into compression molded sheets with a Carver hydraulic press model 4128 (USA). The sheet formation was conducted with a mold having dimensions of 229 × 229 × 0.60 mm^3^ at 180 °C, with a 4000 psi holding pressure for 5 minutes before cooling to room temperature within 8 minutes. The compression molded sheets had a thickness of 0.63 (±0.026) mm.

### Characterization

#### Abrasion resistance

To determine the wear properties of the composites, abrasion testing was conducted conforming to ASTM G195-13a. A rotary platform abraser model 5135 (Taber ind.) was used with vacuum attachment to remove abraded particles. The parameters were set at 72 cycles per min, 500 g mass per wheel, and a CS-10 grit size wheel having 1420 abrasive particles per cm^2^. Initial sample masses and thicknesses were measured for a single specimen of each type and after subsequent 100-cycle abrasion runs, up to a total of 500 cycles. The volume loss of the entire sample and percent thickness reduction from the four measurement points 90° offset were then plotted. For analysis purposes the densities of the PLA, PLA/talc, PLA/BC, and PLA/BC_24 h_ samples were measured with an Alfa Mirage electronic densimeter MD-300S, resulting in values of 1.25, 1.33, 1.27, and 1.28 g cm^3^, respectively.

#### Barrier properties

The oxygen barrier performance of the polymeric sheets was tested using a Mocon OX-TRAN model 2/21 (USA), following ASTM D3985-17. Measurements were conducted at 23 °C with 0% relative humidity and a film area of 50 cm^2^. The oxygen transmission rate (OTR) (cm^3^ m^−2^ d^−1^ atm^−1^) was calculated from the triplicate testing of the samples, which was then converted into oxygen permeation (cm^3^ mm m^−2^ d^−1^ atm^−1^) by using the following equation:1
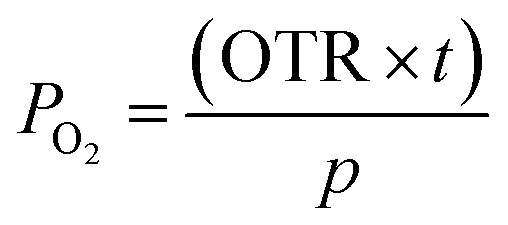
where the *p* is the partial pressure of oxygen, and *t* is the thickness of the sheet.

For the sheets water vapor barrier properties, a Mocon PERMATRAN-W model 3/33 (USA) was utilized according to ASTM E96-16. The analysis was run at 37.8 °C and 100% relative humidity with a film area of 50 cm^2^. Water vapor transmission rate (WVTR) (g m^−2^ d^−1^) was collected from three sample tests, which was converted to water vapor permeation (g mm m^−2^ d^−1^) through the equation:2
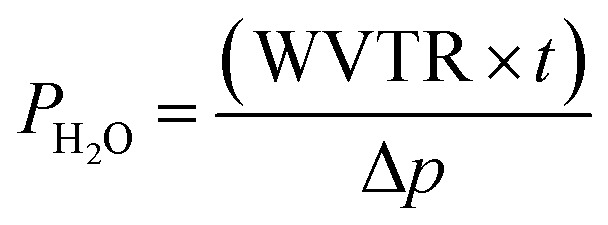
where Δ*p* is the partial water vapor pressure difference between both sides of the sheet, and *t* is the thickness of the sheet.

For the tortuosity factor (*τ*) the quotient was calculated from the total path of diffusing gas (*d*_T_) and the initial thickness of the sheet (*d*_i_) according to Nielsen's 2-dimensional gas diffusion through a polymer equation:^18^3
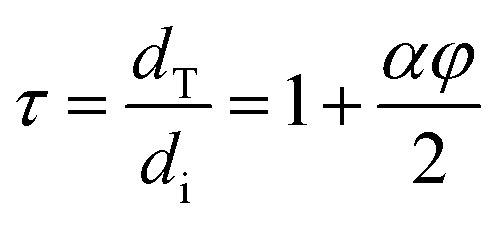
With the variables representing the aspect ratio of the particles, *α*, and their volume fractions, *φ*.

#### Dynamic mechanical analysis

To perform the measurement of the storage modulus for the sheet samples, a dynamic mechanical analyzer (DMA Q800, TA Instruments) was used. This was done with a tension film clamp in the strain control mode following ISO 6721, in which the temperature range was set from 25 to 90 °C with a heating rate of 2 °C min^−1^, a frequency of 1 Hz, a static force of 0.01 N and an oscillating amplitude of 15 μm. The resulting data was analyzed with TA Instruments Universal Analysis 2000 software (ver. 4.5 A).

#### Differential scanning calorimetry

For the determination of the thermal history of the polymeric sheets, runs were carried out on a differential scanning calorimeter (DSC Q200, TA Instruments). Experiments were conducted with ∼8 mg samples with the 1^st^ heating cycle from 40 to 180 °C, followed by cooling cycle down to 0 °C and 2^nd^ heating cycle back to 180 °C. The heating cycles had a rate of 10 °C min^−1^, while the cooling cycle rate was set at 5 °C min^−1^. The glass transition, melting temperatures, and crystallinities were then evaluated through the TA Instruments Universal Analysis 2000 software (ver. 4.5A). Note the value used for 100% crystalline PLA was 93.7 J g^−1^ according to Fischer *et al.*^19^

#### Thermogravimetric analysis

The use of a thermogravimetric analyzer (TGA Q500, TA Instruments) was undertaken to divulge information on the thermal decomposition of the subsequent samples. A heating rate of 10 °C min^−1^ from room temperature to 100 °C was first established and held isothermal for 5 minutes to stabilize the sample before the run began. The data was collected at a ramp rate of 20 °C min^−1^ from 100 °C to 600 °C. The analysis of the resultant TGA curves were performed with the TA Instruments Universal Analysis 2000 software (ver. 4.5A).

#### Flammability testing

To analyze the combustion rate of the samples the UL94 standards were followed in an R.B. Atlas HVUL2 horizontal vertical flame chamber. Initial testing was conducted in accordance with ASTM D635-18 for the horizontal burning (HB) rates. For polymeric specimens with less than 3 mm thickness, the linear burning rate must not exceed 75 mm min^−1^ to be classified as HB, otherwise the samples were marked as having a ‘non-rating’ (NR), as they did not comply with the requirements due to being too flammable. For any sample that the flame front did not reach or pass the 25 mm reference mark to begin timing the burning progression, ASTM D3801-10 for vertical (V) burning classification was conducted. The designated category was then determined based on the afterflame, afterglow times and cotton indicator results. A total of three and five samples were used for the average of the horizontal and vertical burning tests, respectively.

#### Scanning electron microscopy (SEM)

The topography of the worn sheet samples was imaged with a Phenom ProX scanning electron microscope (Phenom-World BV). The images were captured at an accelerating voltage of 10 kV at a magnification of 1000× after pre-coating with gold using a Cressington 108 sputter coater and placed on a charge reduction sample holder to avoid charging.

#### Rheology

For the rheological characterization of the polymer composites, a modular compact rheometer (MCR302, Anton Paar) with a convection temperature device (CTD 450) was employed having Rheoplus software (ver. 3.61). The setup included a 25 mm diameter parallel-plate geometry with a 1 mm gap separation under continuous nitrogen gas purge at a temperature of 180 °C. A dynamic frequency sweep was conducted from 1000 to 0.01 Hz having a shear strain of 1%. The data was also analyzed in terms of the continuous edge preserving regularization (EPR) relaxation time spectrum method.

## Results and discussion

### Abrasion resistance

**Fig. 1:**
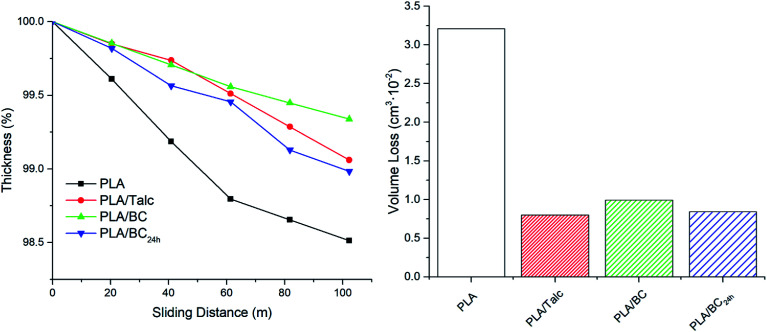
Thickness reduction over sliding distance and total volume loss after abrasive testing of PLA composites.

The effect of the three particulate additives on the abrasive wear behavior in the PLA biocomposites were analyzed. From Fig. 1 it is observed that the addition of the particulate material provides an advantage in minimizing the wear of the surface. With a constant sliding velocity of 0.245 m s^−1^ for the abrading wheels, there is an evident initial thickness reduction up to a 60 m sliding distance for the neat PLA before reaching a more gradual slope consistent with the composite samples. These losses in thickness over sliding distance are a direct result of material removal with progressive scraping of the surface layer. However, after longer periods of sliding the friction coefficient is reduced at the contact location caused by a localized temperature increase and enhanced flexibility in the polymer that gives rise to less thickness loss per distance for PLA.^20^

Next, when regarding the volume loss for the complete abrasion run, the PLA shows more than double that of the talc and biocarbon composites. All three additives were able to impede the wear of the polymeric surface with up to only 1% thickness loss over the course of the sliding distance and less than 0.01 cm^3^ in volume loss. The wear resistance of fillers in general can be attributed to several predominant factors including their hardness, stiffness, compressive strengths and there adhesion to the matrix.^21^ Therefore, that is the reasoning behind the reduced abrasive wear of the composites over the neat PLA, as the fillers have high stiffness and good dispersion throughout the polymer matrix. At larger sliding distances, the PLA/BC had the least thickness loss which may be a result of the larger particles impeding the abrasive wear on the sample surface acting as micron-sized speed bumps.

**Fig. 2:**
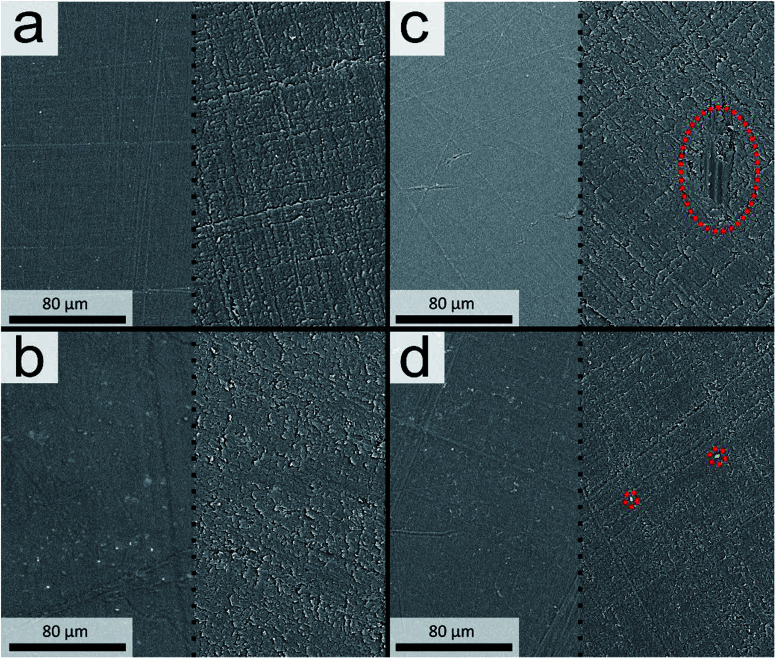
The surfaces of PLA (a) before (left side) and after (right side) abrasion testing and corresponding composites with (b) talc, (c) hammer milled biocarbon [≤400 μm] and (d) 24 h ball-milled biocarbon [<5 μm].

The topographies of the PLA sheet surfaces before and after abrasive testing are depicted in Fig. 2. For all the PLA composite worn surfaces there is material removal from the grinding of the smooth surface in the form of the micro-cutting mechanism. For the neat PLA there is visible signs of deep troughs and grooves that have been chiselled along the surface in the direction of the abrasive grit wheel. Alternatively, in the case of the composites, the surfaces are less disrupted with shallower channels, fine scratches and non-uniform wear, that is a characteristic of augmented wear resistance. Note, the biocarbon PLA-composites have visible particles along portions of the surface after the abrasive testing that remain imbedded within the sample matrix, highlighted in Fig. 2, while the talc particulates are not readily distinguishable.

### Barrier properties

**Fig. 3:**
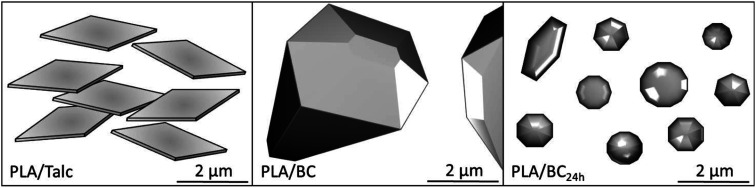
Schematic of the PLA composites.

The barrier properties of the PLA biocomposite sheets were measured for both the oxygen and water vapor transmission. Table 1 shows the results for each sample along with the calculated path of diffusion for the gas through the individual samples and subsequent tortuosity factors. As for the oxygen barrier performance of the PLA with addition of talc there was a reduction in the permeability of the gas by 23% over the neat PLA. While, in the case of the biocarbon composites the opposite was observed with an increase in permeability of 14 to 15%. The observed variation in the oxygen permeability between the talc and biocarbon is primarily a result of the shape, orientation and size of the particles. For the talc, due to the platelet structure it forms an intricate maze that the oxygen must transverse through within the PLA matrix before it reaches the opposite side of the sheet. As for the biocarbon, it does not provide a major blockade for the diffusion of the gas through the PLA as their remains a vast amount of space containing just the PLA matrix throughout the entirety of the thickness of the sheet. This is exemplified in the small differences of 10 to 30 nm calculated for the total diffusion path of the gas through the biocarbon containing sheets over the neat PLA, with the talc having a significantly larger difference of 150 nm of additional distance. Likewise, this results in a tortuosity factor that is 23% larger for the PLA/talc composite rather than the 1–4% increments determined for the biocarbon-filled PLA samples. Another factor that resulted in the reduction of barrier performance for the biocarbon composites is due to the presence of voids and small pores/air pockets the biocarbon particles contain that will increase the transmission rate of the gas and the imperfections throughout the sheet caused by the irregular shapes of the biocarbon. As a visual aid, Fig. 3 is an illustration of the three PLA composites under investigation. SEM images of the cross-sections of the composite samples are also provided in the ESI Fig. S1.† Here the talc provides a barricade to all gases diffusing through the PLA sheet restricting the ease at which it maneuvers through the matrix, unlike the biocarbon and 24 hour milled biocarbon that contain gaps that allow free passage from one side of the sheet to the other.

**Table 1:** Oxygen and water vapor permeability of the PLA composites and their diffusion path length with tortuosity factors

Sample	Oxygen permeability at 23 °C and 0% RH (cm^3^ mm m^−2^ d^−1^ atm^−1^)	Water vapor permeability at 38 °C and 100% RH (g mm m^−2^ d^−1^)	Total path of diffusing gas (μm)	Tortuosity factor
PLA	737 ± (39)	1683 ± (69)	0.63	1.00
PLA/talc	566 ± (9)	1250 ± (67)	0.78	1.23
PLA/BC	850 ± (25)	1958 ± (57)	0.64	1.01
PLA/BC_24 h_	838 ± (17)	1684 ± (27)	0.66	1.04

When analyzing the water vapor barrier performance, a similar trend is observed for the talc and biocarbon composites. The differences may arise from the oxygen moieties present in the biocarbon as observed through FTIR in previous work.^22^ These functionalities including hydroxyl, carboxyl and carbonyl groups, will provide increased affinity to the water vapor due to its hydrophilicity. For the ball-milled biocarbon the resultant permeability was not altered from the PLA unlike the 16% increase for the larger size range biocarbon. Though in the case of talc, its impermeable characteristic and torturous path from its distribution within the PLA matrix make the water vapor permeability less than the neat PLA by a 26% reduction.^23^

In other literature, the crystallinity of the poly(lactic acid) also plays a role in the permeability of the material.^24^ As it is common for the permeability to be reduced with increased crystallinity as the gas travels through the amorphous regions within the polymer matrix. Therefore, the crystallinity of the PLA composites was also characterized through DSC as shown in Table 2. The only noticeable variation is between the neat PLA and that of the composites, with the crystallinity increasing 2–3%. This small change in crystallinity, that is comparable between all filled particles, showcases that the particles themselves play a vital role in the permeability differences measured when crystallinity is not high. The glass transition and melting temperature of these composites were also not affected by the addition of the fillers.

**Table 2:** Glass transition temperature, melt temperature and crystallinity of PLA sheets from DSC

Sample	*T*_g_ (°C)	*T*_m_ (°C)	*χ* (%)
PLA	63 (±2.0)	150 (±0.04)	0.3 (±0.35)
PLA/talc	63 (±1.6)	152 (±0.72)	2.7 (±0.48)
PLA/BC	61 (±2.1)	154 (±0.13)	3.3 (±0.89)
PLA/BC_24 h_	63 (±1.9)	152 (±1.65)	2.0 (±0.42)

### Dynamic mechanical analysis

**Fig. 4:**
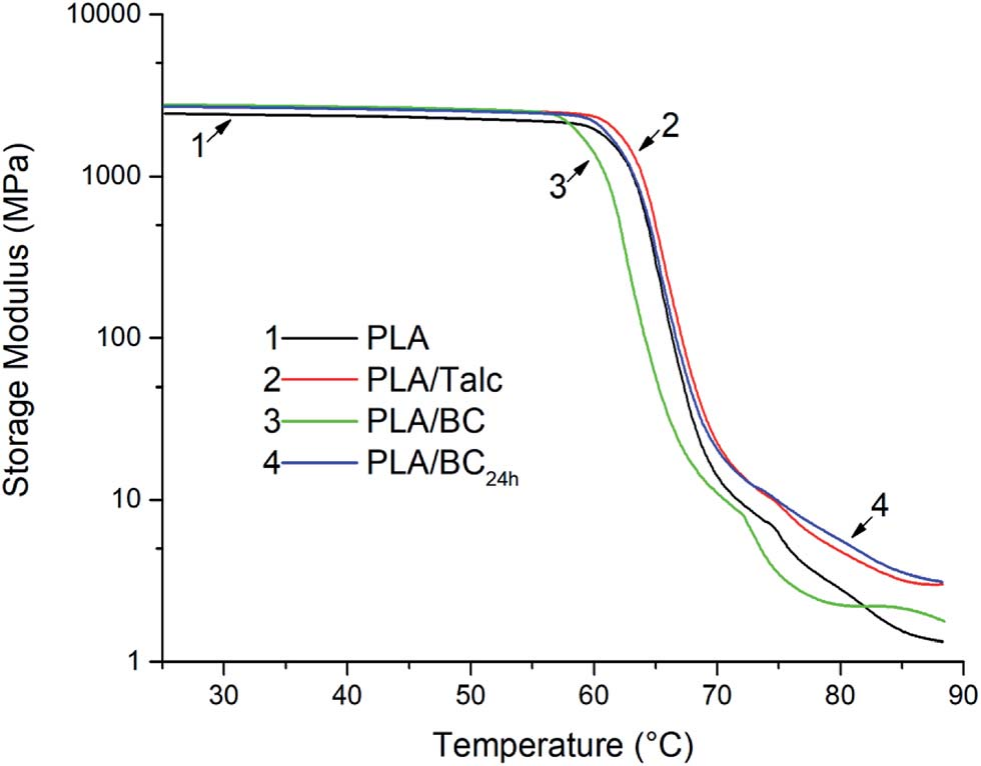
Storage modulus of the PLA composite sheets.

The resultant storage modulus (*E*′) of the PLA composites which experienced dynamic-mechanical testing are displayed in Fig. 4. These results highlight the differences *E*′ undergoes for the PLA with respect to temperature. For the neat PLA sample, *E*′ is approximately 2400 MPa at 25 °C, while the composite samples containing talc or biocarbon have an *E*′ of ∼2700 MPa. The specific enhancements of the samples at 25 °C are 10%, 13% and 10% for PLA/talc, PLA/BC, PLA/BC_24 h_, respectively for the storage modulus. This minor augmentation is a result of the stiff particles present in the samples. Up to 55 °C, just below *T*_g_, the composites retained their *E*′ with percent increases between 12 and 14%. The elastic properties of the PLA are affected by the inclusion of the particles.^25^ Once the samples have reached the glass transition temperature they all begin to decrease in storage modulus at a similar rate, though the PLA/BC has an earlier onset over that of the other three samples by 2 °C which matches with the lower *T*_g_ measured from the DSC. The lower *T*_g_ of the PLA/BC compared to that of the other samples is suggested to be a result from the degradation effects of the BC on the PLA which is supported by the reduced onset degradation temperature in TGA and relaxation time spectrum in the rheology studies provided in the next sections. At high temperatures above 80 °C, *E*′ is greater for all filled samples relative to the PLA. The soft matrix at these high temperatures is reinforced by the talc or biocarbon particles through the restriction of the polymer chains movement.^26^ This enhancement results in *E*′ at 85 °C having an increase for PLA/talc, PLA/BC, and PLA/BC_24 h_ of 106%, 38%, and 129%, respectively. The larger *E*′ for both the talc and ball milled biocarbon is from the greater dispersion and overall larger number of particles that are present in the composite as compared to the larger particle size biocarbon. For the tan *δ* the same trend is observed with the PLA/BC tan *δ* peak occurring at a temperature 2 °C lower than the rest of the samples. Along with this the three filled samples all have a minor reduction in their tan *δ* values as the composites act more elastically in their response with the hindered molecular motion from polymer chain and particle interactions. Overall, the thermomechanical stability based on the increased storage modulus was enhanced with the talc and biocarbon acting as reinforcements. The smaller fillers tended to work better as reinforcing agents, likely due to finer distribution of particles and reduced degradation effects observed in TGA and rheology studies discussed later.

### Flammability

**Fig. 5:**
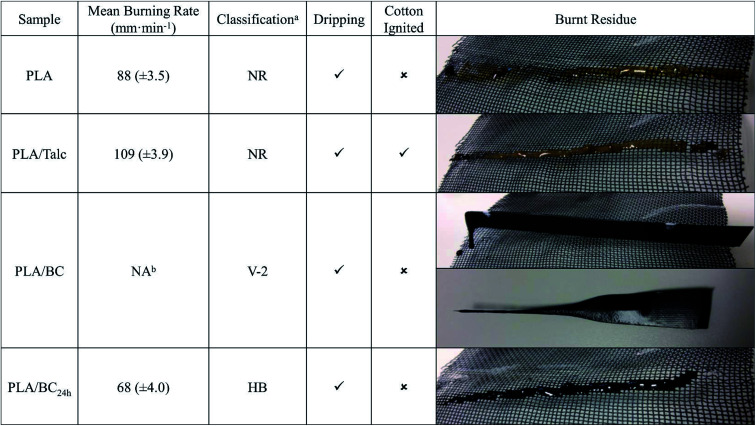
The Burning rates, classification and resultant PLA samples after flammability testing. ^*a*^Specimen thicknesses of 0.63 mm; NR ≡ non-rating; less than 75 mm min^−1^ burning rate allows classification of HB; ^*b*^flame front did not reach the 25 mm reference mark as seen in upper image (NA ≡ not available), therefore vertical burning test evaluated shown in lower image.

There are several methods to determine the flame-retardant characteristics of thermoplastic materials that differ in the factors being investigated. These fire related variables include such measurements as the burning rates, afterglow times, ignition capabilities, quantity and rate of heat released during burning and flame spread.^27^ Therefore, to determine the impact of the particulate fillers on the burning properties of the PLA, the samples were subjected to the UL94 HB testing as a preliminary investigation, with the results shown in Fig. 5. The table summarizes the qualitative and quantitative information obtain from the burn tests, including the mean burning rate, classification, the observational information and images of post-burning samples. In the case of the composites, the talc and biocarbon are primarily acting as filler to dilute the amount of polymer present. As such, the quantity of thermoplastic material present during the decomposition will be reduced which will lower the fuel support rate to the burning zone. However, talc has been found to cause a reduction in the fire retardancy of composites due to the formation of magnesium moieties that catalyze the degradation and is not able to provide a benefit when less than or equal to 20 wt% loadings.^28^ Similarly, both the neat PLA and PLA/talc samples failed to pass the UL94 test, as both burning rates exceeded 75 mm min^−1^ in the horizontal setup. Along with this, the material was easily ignited and the propagation of the fast-moving flame lead to the polymer stretching and dripping, with the talc containing sample also igniting the cotton.

As for the ball-milled biocarbon, the burning rate was reduced within the limit of the HB classification, due to the biocarbon providing sufficient hindrance to the propagating fire. The PLA/BC_24 h_ did have dripping occur which was also found with the neat PLA, but both these samples had their drips extinguished before reaching the cotton from the increased timeframe of each drop. In contrast, the PLA/BC sample did not propagate the flame along the horizontal direction, as the material would melt and drip too quickly for the remainder of the sample to ignite. Therefore, the PLA/BC was subjected to the vertical burning test, and the same observation occurred with the material tending to drip rather than combust. Therefore, this sample fell under the category of V-2, for this thickness, as there were still drips occurring from the sample, though they were not inflamed and did not ignite the cotton. Note that the burning stopped immediately after removal of flame, even though the standard mentions within 30 seconds for this classification.

Part of the increased flame retardancy associated with the biocarbon-filled samples may arise from the carbonaceous material providing a barrier to the leading edge of flame front. As it is known that carbonized (char) surface layers can form during burn tests that will act as an insulating layer, limiting heat transfer, reducing combustion and preventing the polymer from direct contact with the flame.^29^ This layer is able to protect the underlying material from oxygen diffusion and the volatile degradation products from fueling the combustion. Though in the case of these samples, the thickness is relatively small allowing the flame to propagate *via* heat transfer from the sides of the sample rather than the central region, reducing the effectiveness of the char layer. Though, due to all the samples having the behavior of burning with drips the utilization in applications such as electrical parts will be limited.^30^

To investigate further, the variation in the degradation characteristics of the PLA samples, thermogravimetric analysis was performed. Commonly, PLA is primarily known for thermal degradation associated with thermo-hydrolysis from the presence of residual water, zipper-like depolymerization from trace catalyst, thermo-oxidative degradation with random main-chain scission, intermolecular transesterification forming monomer and oligomers of low molecular weight lactides, residual acidity, and metal impurities.^31^

**Fig. 6:**
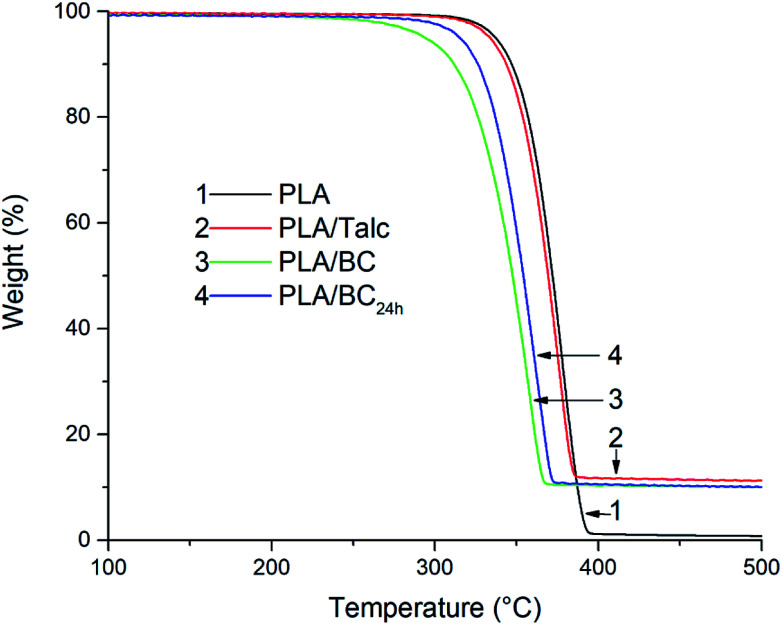
The degradation characteristics of PLA composite sheets using TGA.

As seen in Fig. 6 the thermal stability of the PLA samples has been compared between the neat PLA and the composites. The talc and biocarbon fillers are stable within the temperature range under investigation, from room temperature to 600 °C. Yet, there remains variation in the effect fillers may have on the degradation of the thermoplastic polyester, both positive with stabilizing capabilities or negative in augmented degradation. Though there is difficulty in predicting the thermal behavior based on the additives alone, the factors such as particulate size, shape, purity, and dispersion come into play along with any surface treatments, intrinsic thermal properties, and existence of any impurities or detrimental residues.^32^

As for the PLA and its composites under investigation there resides a noticeable difference between the talc and biocarbon fillers. Where, both PLA and the PLA/talc have a similar thermal stability with an onset of degradation temperature (5% weight loss) occurring at 337 °C and 334 °C, respectively. While the 24 hour milled biocarbon composite has an onset degradation temperature approximately 20 °C below at 315 °C, and the PLA/BC is even less at 294 °C or ∼40 °C below the neat PLA. Likewise, the temperature of maximum degradation rate has the same trend with PLA > PLA/talc > PLA/BC_24 h_ > PLA/BC (378, 376, 362, and 356 °C, respectively). These observed differences in the thermal stability of the PLA composites can be explained in part by the higher moisture content of 1.5% in the biocarbon before processing over the talc's 0.5% moisture from entrapped water in the pores of the biocarbon, causing thermo-hydrolysis of the PLA chains along with the presence of any oxidative degradation from air captured in the porous structures. While the larger biocarbon particles have less uniformity over the ball-milled biocarbon which may impart additional degradation from shearing, and greater water retention in the pores with diffusion for the larger particles being more difficult with longer distances to travel over the smaller particle variant, which will also promote degradation of PLA. This aspect is further investigated in the next section *via* rheology.

### Rheological characteristics

**Fig. 7:**
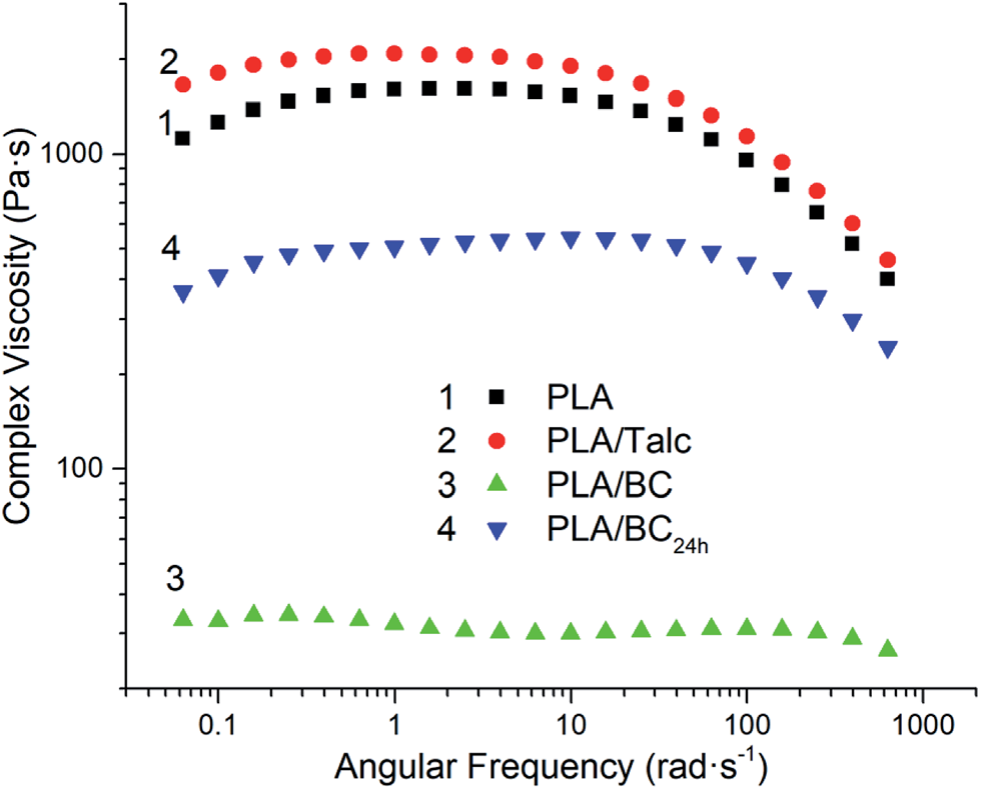
Dependence of complex viscosity *versus* angular frequency of the PLA composites.

**Fig. 8:**
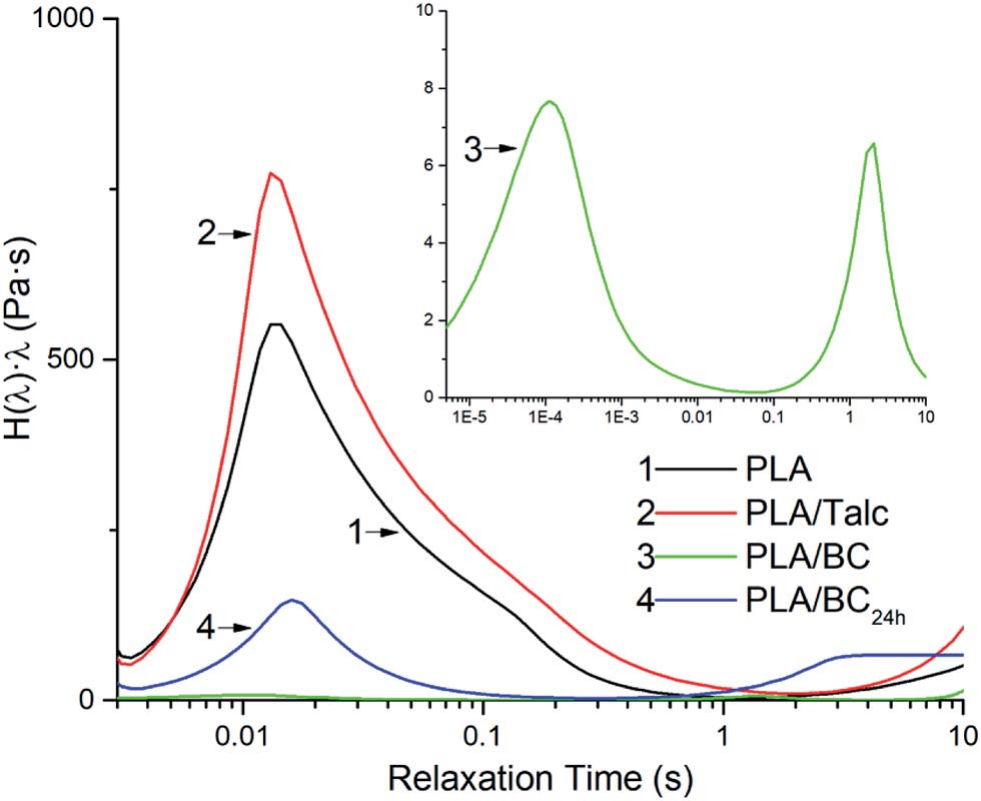
The continuous enhanced relaxation time spectrum of the PLA composites obtained by frequency sweeping at 180 °C.

The effect of the fillers on the complex viscosity and relaxation spectrum of PLA is depicted in Fig. 7 and 8, respectively. The neat PLA exhibited constant shear-viscosity in low frequency because of the absence of chain entanglement. With the introduction of particle fillers, the relaxation spectrum and viscoelasticity properties have been reported to be influenced by polymer–particle interactions, hydrodynamic effects and particle–particle interactions.^33,34^ Normally, the incorporation of filler into a polymer matrix enhances the storage modulus and shear viscosity of polymers because of the stronger filler networks, especially above the critical concentrations of the fillers.^35^ The increased viscosity of PLA/talc composites reflects the effects of strong filler–filler interactions and its interactions with the PLA chains because of the small particle size of 2 μm and good distribution of the talc filler. Different from the PLA/talc composites, the PLA/biocarbon composites show lower complex viscosity compared to neat PLA. The viscosity of PLA/BC without post-milling is only 10 Pa s *vs.* 1000 Pa s for the neat PLA, reflecting the poor particle–polymer interactions. This is probably caused by the opposite polarity between PLA and biocarbon, large particle size of the BC and the degradation of PLA from biocarbon during the melt processing.

As for the intrinsic characterization of the polymer viscoelasticity properties, the relaxation time spectrum can be applied to distinguish the relaxation model of different units, from polymer chains to filler network.^36^ The continuous weighted relaxation time spectrum *H*(*λ*) is obtained from the dynamic modulus in rheology testing^37^ and edge preserving regularization method in the Rheoplus analysis software as eqn (4a) and (4b).4a
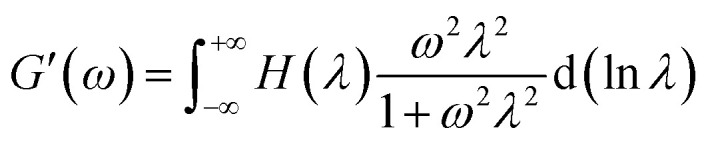
4b
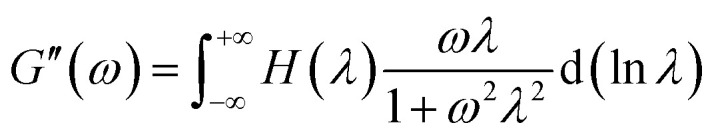
where *G*′(*ω*) and *G*′′(*ω*) are storage and loss modulus of the melt, *ω* is the angular frequency, and *λ* is the relaxation time. The obtained relaxation spectrum is plotted in Fig. 8.

As a stiff network, the introduction of filler normally enhances the relaxation strength of the polymer matrix, and the relaxation time of the polymer chains is also increased because of the limitation in mobility brought on by the polymer–filler interactions.^38^ As a brittle polymer without any branching chains, the longest relaxation time of PLA is rather low with a value of ∼0.02 s. From the relaxation time spectrum of PLA composites with different kinds of fillers shown in Fig. 8, it can be found that talc enhances the relaxation strength of the PLA chains as a traditional filler used in polymer engineering. The addition of biocarbon, however, decreases the relaxation strength of the PLA chains and along with this the longest relaxation time of PLA is decreased with the additional of BC. The decreased relaxation strength of PLA indicates the poor interaction between the PLA and biocarbon resulted from the different polarity of PLA and biocarbon, and the melt entanglement of PLA chains has been weakened because of the mobility of the biocarbon filler networks in the matrix. In the relaxation time spectrum of PLA/BC composites, two weak peaks appear from 0.0001 to 10 s and the relaxation peak of PLA chains at 0.02 s disappears. It is suggested that the chain structures of PLA have been changed in the presence of BC during melt processing. Considering the decreased viscosity and rather low relaxation strength, the low relaxation peak is ascribed to the degraded PLA chains while the high peak is the relaxation of the BC filler networks under shearing because of its rather big particle size (∼20–75 μm). The rheological studies show that the PLA-biocarbon particle interaction is very poor because of the hydrophilicity of biocarbon; and the degradation of PLA is promoted by the biocarbon particles during melt processing. Therefore, the hydrophilic nature of biocarbon endows this filler with high water absorption which increases the thermal degradation of PLA, a biodegradable polymer which is sensitive to the moisture contents,^39^ during the melt processing.

## Conclusions

In this presented research, the barrier properties, wear performance and flammability characteristics of particulate filled PLA biocomposites were examined. These measurements contrasted the performance of biocarbon filler relative to talc, a commonly used composite filler. With packaging applications in mind, the transmission rates for both oxygen and water vapor demonstrated that the talc provided a tortuous path that reduced the permeability of the compression molded sheet. While on the other hand, those containing the biocarbon constituents reduced the oxygen barrier and provided no change or inferior performance with water vapor based on the particulate size. This renders biocarbon unsuitable for use alone in the adaption of barrier-oriented products.

Next, when regarding the wear behavior, all fillers augmented the abrasive resistance of the PLA samples due to their stiffness, with less thickness and volume loss, establishing that the composites have improved tribological properties. Therefore, the modification of durable composites with biocarbon may be feasible as the frictional wear in such material will be limited.

Lastly, the flammability attributes of the composites were found to show that the biocarbon is a superior filler than the talc in reducing the burning rate. This enhancement with filler arises in part from the charring interference biocarbon creates during combustion and the obstruction with the flow of the polymer from the irregular size and shape. Yet, the occurrence of drips causes all samples including the PLA alone to be inadequate for electronic or high heat applications without additional fire-retardant additives or treatments.

In conclusion, the results provided herewith develop a standard baseline for further PLA-biocarbon composite optimization through secondary additives for select applications.

## Conflicts of interest

There are no conflicts to declare.

## Supplementary Material

RA-009-C9RA00034H-s001
