# Correction: Comparative study of the extrinsic properties of poly(lactic acid)-based biocomposites filled with talc *versus* sustainable biocarbon

**DOI:** 10.1039/d1ra90158c

**Published:** 2021-10-14

**Authors:** Michael R. Snowdon, Feng Wu, Amar K. Mohanty, Manjusri Misra

**Affiliations:** School of Engineering, Thornbrough Building, University of Guelph 80 South Ring Rd E Guelph Ontario N1G 1Y4 Canada mmisra@uoguelph.ca mohanty@uoguelph.ca snowdonm@uoguelph.ca; Bioproducts Discovery & Development Centre (BDDC), Department of Plant Agriculture, Crop Science Building, University of Guelph 117 Reynolds Walk Guelph Ontario N1G 1Y4 Canada fengwu@uoguelph.ca

## Abstract

Correction for ‘Comparative study of the extrinsic properties of poly(lactic acid)-based biocomposites filled with talc *versus* sustainable biocarbon’ by Michael R. Snowdon *et al.*, *RSC Adv.*, 2019, **9**, 6752–6761, DOI: 10.1039/C9RA00034H.

The authors regret that the values given for oxygen and water vapor permeability in Table 1 were incorrect in the original article. The correct version of the table is shown here.

**Table 1:** Oxygen and water vapor permeability of the PLA composites and their diffusion path length with tortuosity factors

Sample	Oxygen permeability at 23 °C and 0% RH (cm^3^ mm m^−2^ day^−1^ atm^−1^)	Water vapor permeability at 38 °C and 100% RH (g mm m^−2^ day^−1^)	Total path of diffusing gas (μm)	Tortuosity factor
PLA	7.37 ± (0.39)	16.83 ± (0.69)	0.63	1.00
PLA/talc	5.66 ± (0.09)	12.50 ± (0.67)	0.78	1.23
PLA/BC	8.50 ± (0.25)	19.58 ± (0.57)	0.64	1.01
PLA/BC_24 h_	8.38 ± (0.17)	16.84 ± (0.27)	0.66	1.04

The Royal Society of Chemistry apologises for these errors and any consequent inconvenience to authors and readers.

## Supplementary Material

