# Supplementary material for: Comparative study of the extrinsic properties of poly(lactic acid)-based biocomposites filled with talc versus sustainable biocarbon
Source: RSC Adv. 2019 Feb 26;9(12):6752–61. doi: 10.1039/c9ra00034h (PMC9061034; doi:10.1039/c9ra00034h)
Supplement: RA-009-C9RA00034H-s001 [file RA-009-C9RA00034H-s001.pdf]

Electronic Supplementary Material (ESI) for RSC Advances.
This journal is © The Royal Society of Chemistry 2019

Comparative Study of Extrinsic Properties of Poly(Lactic Acid)-Based Biocomposites Filled With Talc Versus Sustainable Biocarbon

Michael R. Snowdon [a,b], Feng Wu [b], Amar K. Mohanty [a,b*], Manjusri Misra [a,b*]

[a]School of Engineering, Thornbrough Building, University of Guelph, 80 South Ring Rd E, Guelph, Ontario, Canada, N1G 1Y4; [b]Bioproducts Discovery & Development Centre (BDDC), Department of Plant Agriculture, Crop Science Building, University of Guelph, 117 Reynolds Walk, Guelph, Ontario, Canada, N1G 1Y4

Supplementary Results:

From the cross-sectional images of the PLA composite sheets, the dispersion of the fillers can be seen. The talc shows random dispersion throughout the matrix with layer-by-layer agglomeration, as seen by the larger white particles present in Figure S1. As for the PLA/BC sample the biocarbon fragments are seen protruding from the surface in several directions that are not well dispersed within the PLA matrix. As for the 24-hour ball-milled biocarbon the particles are quite uniformly scattered within the polymeric matrix but have a larger interparticle spacing than that of the talc-filled sample. These variations in particle dispersion are responsible in part for the barrier performance of the composites as the talc provides the most tortuous path.

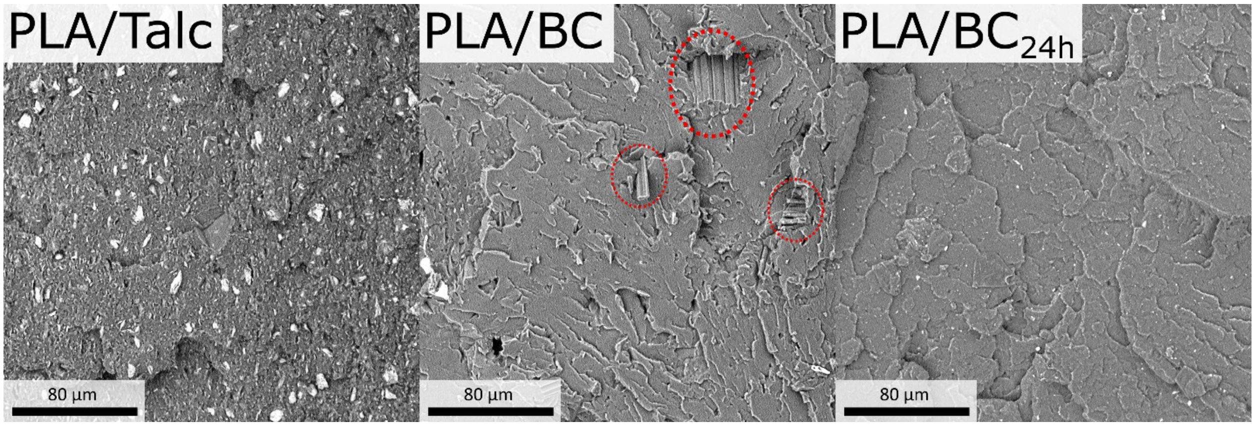

Figure S1. SEM cross-section of the PLA composites.
